# Peptides Targeting the Interaction Between Erb1 and Ytm1 Ribosome Assembly Factors

**DOI:** 10.3389/fmolb.2021.718941

**Published:** 2021-09-01

**Authors:** Lidia Orea-Ordóñez, Susana Masiá, Jerónimo Bravo

**Affiliations:** Department Genomics and Proteomics, Instituto de Biomedicina de Valencia, Spanish National Research Council (CSIC), Valencia, Spain

**Keywords:** structural-guided peptide selection, interference peptides, targeting ribosome biogenesis, protein–protein interactions, Erb1/Ytm1 complex

## Abstract

Ribosome biogenesis is an emerging therapeutic target. It has been proposed that cancer cells are addicted to ribosome production which is therefore considered a druggable pathway in cancer therapy. Cancer cells have been shown to be more sensitive to inhibition of the ribosome production than healthy cells. Initial attempts of inhibiting ribosome biogenesis have been focused on the inhibition of transcription by targeting RNA Pol I. Despite being a promising field of research, several limitations have been identified during the development of RNA Pol I inhibitors, like the lack of specificity or acquired resistance. Ribosome biogenesis is a multistep process and additional points of intervention, downstream the very initial stage, could be investigated. Eukaryotic ribosome maturation involves the participation of more than 200 essential assembly factors that will not be part of the final mature ribosome and frequently require protein–protein interactions to exert their biological action. Using mutagenesis, we have previously shown that alteration of the complex interface between assembly factors impairs proper ribosome maturation in yeast. As a first step toward the developing of ribosome biogenesis inhibitory tools, we have used our previously solved crystal structure of the *Chaetomium thermophilum* complex between the assembly factors Erb1 and Ytm1 to perform a structure-guided selection of interference peptides. The peptides have been assayed *in vitro* for their ability to bind their cellular partner using biophysical techniques.

## Introduction

The synthesis of ribosomes in eukaryotes is a sophisticated and energy-demanding process requiring the participation of more than 200 assembly factors that will not be part of the final mature ribosome although are required for a correct ribosome biogenesis. The initial steps of ribosome assembly take place in the nucleolus where rRNA is transcribed by RNA polymerase I (RNA pol I) [reviewed in (Baßler and Hurt, 2019)] under the regulation of both tumor suppressor genes (including p53, Rb, and Arf) and oncogenes (including MYC, MAPK/ERK, PI3K, and AKT) (Montanaro et al., 2008; Morcelle et al., 2019). Increased ribosome biogenesis is important for cell transformation or tumorigenesis and it is assumed as a general trend in cancer cells that need to make extra ribosomes in order to produce more proteins to sustain uncontrolled cell division (Orsolic et al., 2016) (Truitt and Ruggero, 2016). Interestingly, it has been shown that cell proliferation can be blocked by inhibiting the production of new ribosomes since impaired ribosome biogenesis induces a checkpoint control that prevents cell cycle progression (Volarević et al., 2000). It is therefore not surprising that biogenesis of the ribosomes has been accumulating growing attention as a potential new therapeutic target.

Initial attempts to specifically target ribosome biogenesis have been focused on the downregulation of RNA pol I. Several recently described small molecules like CX-5461, CX-3543, BMH-21, or CID-765471 are now providing evidence that inhibition of ribosome biogenesis by targeting transcription of ribosomal DNA has a promising therapeutic potential (Bywater et al., 2012; Colis et al., 2014). However, lack of specificity and acquired resistance suggest that a new generation of Pol I inhibitors should be developed. Moreover, the only inhibitor that reached clinical trials has shown additional activities contributing to its toxicity profile and resistance, and therefore, additional points of intervention during the ribosome maturation process should be explored apart from the inhibition of transcription (Catez et al., 2019; Ferreira et al., 2020). Despite the fact that the complexity of the process yields a large repertoire of potential targets, only very few chemical inhibitors of ribosome biogenesis are known so far (Awad et al., 2019). Apart from the lack of molecular details in the process, the main reason for this limited number of inhibitors is that the complex ribosome biogenesis pathway is orchestrated by a wide range of macromolecular interactions sequentially coordinated to promote the correct ribosome maturation. Ribosome assembly factors frequently require protein–protein interactions in order to exert their biological actions (Li et al., 2017) and these have been traditionally hard to target by small molecule drugs given the absence of grooves or binding pockets in the interaction surface.

Nop7, Erb1, and Ytm1 are assembly factors that form a discrete heterotrimer that can be detected in isolation from the pre-ribosomal particles (Tang et al., 2008). The so-called Nop7 complex (PeBoW in mammals) is essential for a correct maturation of the ribosomal 60S subunit. The three components guarantee the correct maturation of 5’ end of 5.8S rRNA, thus facilitating its association with 25S rRNA in the mature ribosome (Granneman et al., 2011). We have previously reported the structural resolution of the *Chaetomium thermophilum* Erb1/Ytm1 complex (Marcin et al., 2015; Wegrecki et al., 2015). The crystal structure shows Ytm1 bound to the carboxy-terminal portion of Erb1. Integrity of the heterotrimer assembly is essential for exerting its biological action. In fact, we previously showed that compromising the stability of the Erb1/Ytm1 interaction has an effect on proliferation in yeast (Wegrecki et al., 2015).

Using our previous Erb1/Ytm1 structure, we have performed a structure-guided selection of a set of peptides derived from their sequences and test their ability to bind to their respective cellular partners, Erb1 or Ytm1. Our results open the possibility to obtain peptides with the ability to interfere in the ribosome biogenesis pathway. To our knowledge, this is the first report of peptides designed to target ribosome biogenesis.

## Materials and Methods

### Cloning, Expression, and Purification of Ytm1 and Erb1

Protein expression and purification was carried out following the protocol described in a study by Wegrecki et al. (2015). The *YTM1* gene from *Chaetomium thermophilum* was obtained by total cDNA amplification and cloning in pOPIN-F using an In-Fusion cloning system commercial kit (Clontech). Sf9 insect cells were grown in the Sf900 II SFM medium (Gibco) and were transfected with Ytm1-pOPINF and linearized Ian Jones bacmid (Zhao et al., 2003). Baculovirus generated were amplified and used to induce protein expression for 72 h at 27°C. Erb1 was cloned in a pET28-NKI/LIC 6His/3C vector (from NKI Protein Facility, Amsterdam) by the ligase independent cloning (LIC) method and expressed in *Escherichia coli* (DE3) BL21 CodonPlus (RIPL) using an auto-induction system (Studier, 2005). The 6xHis tagged Ytm1 and Erb1 proteins were purified using a Histrap-HP Ni charged column (GE Healthcare) eluting with a 20–500 mM imidazole gradient. An additional step of a HiTrap Heparin HD column (GE Healthcare) eluting with 0.2–1.5 M NaCl gradient was included for Erb1. A final polish step of size exclusion chromatography (HiLoad 16/60 Superdex 200 column, GE Healthcare) was performed for both proteins that were flash cooled under liquid nitrogen and stored at −80°C until use.

### Biolayer Interferometry Assays

Peptides were commercially obtained from Synpeptide Co., Ltd., Shanghai, China. All experiments have been made in triplicates and include control curves for bait and analyte only.

#### Peptide Interference Experiments

Potential interference peptide samples were evaluated by their ability to interfere the kinetics and affinity of the complex between Erb1 and Ytm1 as measured by Biolayer Interferometry (BLItz, Pall FortéBio Corp.) using Ni-NTA biosensors (FortéBio) and 50 mM HEPES, pH 7.5; 150 mM NaCl; 5% (v/v) glycerol; and 2 mM β-mercaptoethanol (BME) buffer. To set the reference K_D_, an 80 µg/ml solution of Erb1 was immobilized to the biosensor in order to obtain binding curves for increasing concentrations of Ytm1. According to this, an evaluation of Erb1-derived peptides (P1–P3) was performed using 5 µM Ytm1 previously incubated during 30 min at equimolar concentrations of each peptide. New K_D_ values were determined. Similarly, an 80 µg/ml solution of Ytm1 was immobilized to the biosensor in order to obtain binding curves for increasing concentrations of Erb1. 20 µM Erb1concentration was selected for the analyte. Each Ytm1-derived peptide (P4–P6) was evaluated by preincubation of equimolar concentrations with 20 µM Erb1 assayed against immobilized Ytm1 of 80 µg/ml.

#### Peptide Affinity Experiments

BLI was used to determine the affinity between Ytm1 and biotinylated peptides using streptavidin biosensors (FortéBio). The biosensors were hydrated 10 min in the same buffer used on the interference assay, with 0.05% (w/v) bovine serum albumin (BSA). 50 µg/ml of biotinylated peptide was immobilized at the streptavidin biosensor. Increasing concentrations of Ytm1 were used to calculate the K_D_ values using the Blitz Pro 1.2 software using the implemented equations for association and dissociation:

Association phase:

$$\text{y}={\text{R}}_{\text{max}}\;\frac{1}{1+\frac{k_{\text{d}}}{k_{a}*\left[Analyte\right]}}\left(1-e^{-\left(k_{\text{a}}*\left[\text{Analyte}\right]+k_{\text{d}}\right)\text{x}}\right)\;.$$

Dissociation phase:

$$\text{y}={\text{y}}_{0}\;e^{-k_{\text{d}}\left(\text{x}-{\text{x}}_{0}\right)}.$$

$$\text{y}0={\text{R}}_{\text{max}}\;\frac{1}{1+\frac{k_{\text{d}}}{k_{a}*\left[Analyte\right]}}\left(1-e^{-\left(k_{\text{a}}*\left[\text{Analyte}\right]+k_{\text{d}}\right){\text{x}}_{0}}\right).$$

### Differential Scanning Fluorimetry

Thermofluor assays (Pantoliano et al., 2001) were performed on a 7,500 Fast Real-Time PCR System (Applied Biosystems) measuring the gradual fluorescence generated by a 1:1,000 dilution of SYPRO Orange Protein Stain Gel (Supelco, Merck-Sigma) along with 1°C/min temperature increase from 20°C until 85°C.

The samples were mixed containing the protein Ytm1 at 5 µM in 50 mM HEPES pH 7.5, 150 mM NaCl and peptides P1-P3 at 1 mM concentration. Previously, lyophilized P2 and P3 were resuspended in the same Ytm1 buffer and P1 was solubilized in 10 mM Tris (hydroxymethyl)-methylamine (Tris-HCl), pH 8.8 for P1. Data of triplicated experiments were analyzed using GraphPad Prism 5.01 software.

### MicroScale Thermophoresis

Microscale thermophoresis (MST) was used to determine the binding affinity between Ytm1 and P1. Ytm1 was labeled with a Red-NHS second-generation dye kit (100 µl at 10 µM of protein +300 µM dye solution) for 30 min at room temperature in the dark. 5 nM of labeled Ytm1 in 50 mM HEPES pH 7.5, NaCl 150 mM supplemented with 0.1% (v/v) Pluronic F-127 was used for the assay. P1 stock is diluted in 10 mM Tris (hydroxymethyl)-methylamine (Tris-HCl), pH 8.8. Ytm1 and P1 were mixed at 1:1 molar ratio in sixteen serial dilutions (1,230 μM–0.0751 µM) using the same buffer. Measurements were taken on a Monolith NT.115 instrument (NanoTemper Technologies). Similarly biding between Ytm1 and P3 using MST was tested using the same Ytm1 buffer also for P3. Curve fitting and K_D_ calculations every 10 min were analyzed from three independent experiments with MO. Maximum binding was observed at 50 min. Curves were analyzed using Affinity Analysis Software (NanoTemper Technologies)**.**

### Bioinformatics

**FIGURE 1:**
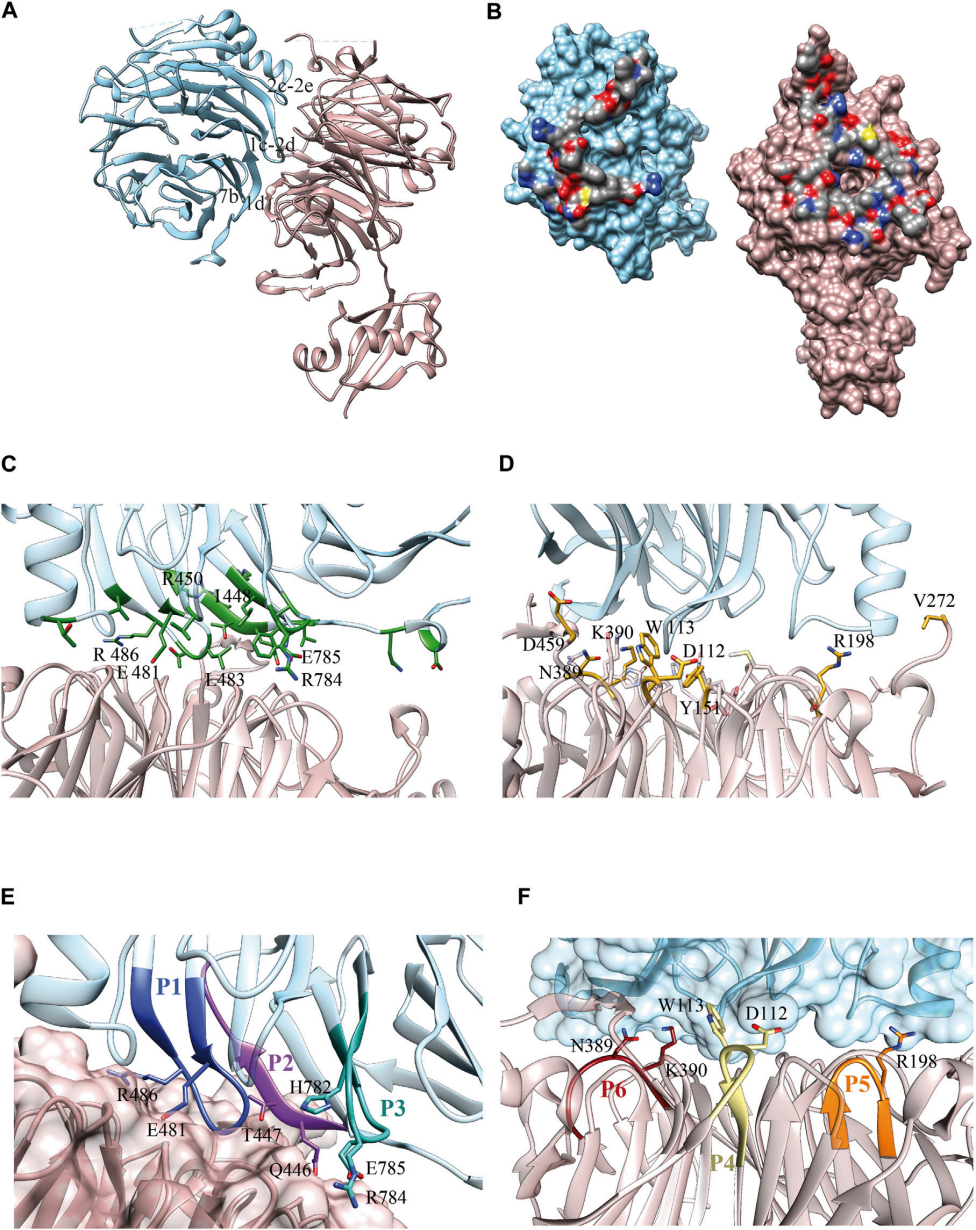
Erb1/Ytm1 interaction. **(A)** Ribbon representation of the heterodimer. Top face of Ytm1 β-propeller in pale pink interacts with Erb1 β-propeller’s side in blue. The main secondary structure-interacting motifs are shown in Erb1 **(B)** Surface representation of the individual components with the interaction areas facing toward the observer. Atoms from residues involved in the interaction are colored by atom, carbon in grey, oxygen in red, and nitrogen in blue. The rest of the surface is colored in blue for Erb1 and pale pink for Ytm1. **(C)** Calculated Erb1 ΔΔG values from in silico alanine scan. Residues from Erb1 with calculated ΔΔG>0.5 kcal/mol are depicted in green. **(D)** Calculated Ytm1 ΔΔG values from in silico alanine scan. Ytm1 residues with calculated ΔΔG>1.0 kcal/mol are represented in yellow. **(E)** Selected peptides derived from Erb1. Each peptide is colored with a different shade of blue and is labeled according to Table 1. Side chains of interacting residues establishing electrostatic interactions are shown. **(F)** Selected peptides derived from Ytm1. The three selected peptides are depicted in brown, yellow, and orange for P6, P4, and P5, respectively (Table 1). Side chains of interacting residues establishing electrostatic interactions are shown.

Buried surface calculations were performed using the protein interfaces, surfaces, and assemblies service PISA at the European Bioinformatics Institute (http://www.ebi.ac.uk/pdbe/prot_int/pistart.html) (Krissinel and Henrick 2007) using PDB ID 5cxb and in silico alanine scanning of the interaction surface was performed using the DrugScorePPI Web Interface (https://cpclab.uni-duesseldorf.de/dsppi/) (Krüger and Gohlke, 2010) only considering polypeptide atoms. ΔG computed for alanine mutants for each 5cxb polypeptide chain is compared to ΔG computed from the wild type complex. Resulting ΔΔG predicts the contribution of a given side chain to the wild type complex stability (ΔΔG = ΔGALAcomplex−ΔGWTcomplex). Interacting atoms between Erb1 and Ytm1 were also analyzed using the 5cxb coordinates program contact in the CCP4 suite (Winn et al., 2011). Molecular graphics in Figure 1 were performed with UCSF Chimera (Pettersen et al., 2004).

## Results

### Interaction Surface Analysis and Peptide Selection

Our Erb1/Ytm1 heterodimer crystal structure (PDB ID 5CXB) (Wegrecki et al., 2015) shows that the central part of the β-propeller of Ytm1 provides a large docking surface for the bottom face of blades 1, 2, and 7 from Erb1 that is additionally held in place by two lateral extensions from Ytm1. The Ytm1 β-propeller top face, away from the N-terminal Ubiquitin-like domain, establishes extensive contacts with the side face of Erb1 C-terminal β-propeller (Figure 1A) with a predominant role of blade 7. The Erb1/Ytm1 heterodimer is mainly maintained by electrostatic forces with some hydrophobic regions also involved in the interaction (Figure 1B). No clear grooves have been detected in the heterodimer interaction surface.

Manual inspection of the interaction surface between Erb1 and Ytm1 combined with buried surface area upon binding calculations using PISA (Krissinel and Henrick, 2007), together with an analysis of the interactions and an alanine scanning of the interacting residues to locate hotspots using DrugScore^PPI^ (Krüger and Gohlke, 2010), revealed several areas of interaction (Figure 1C, Supplementary Table S1, S2). The last β-strand (“1d”) of blade 7 in Erb1 contacts loop “6d-6a” and a long extension that appears between strands “7d” and “7a” of Ytm1 (the knob formed by residues 444–460). A second interaction area involves the entrance of the central tunnel of Ytm1 as a docking site for a loop between strands “1c–2d” from the first blade of Erb1 (481–486) (Figure 1C). The loop contains three well conserved residues: E481, T484, and R486 that establish a network of electrostatic interactions with also conserved amino acids from blades 1, 2, 3, and 7 of Ytm1. We have previously reported the relevance of the second area of interaction since the R486E point mutation decreased the affinity of the interaction by two orders of magnitude without affecting structural integrity. The equivalent mutation in yeast impaired growth in yeast and affected 60S subunit biogenesis (Wegrecki et al., 2015). As indicated by the in silico alanine scanning analysis, this interacting region also suggests a hot spot between Erb1 and Ytm1 (Figure 1C, Supplementary Table S1). Erb1 a-b loop from blade 7 participates in another interaction area with Ytm1 loops from blade 3. Finally an insertion in blade 2 of the β-propeller of Erb1 between 2c and 2e (Supplementary Figure S1) interacts with loops from blades 2 and 3 and an extension between strands 3c and 4d from Ytm1 (Supplementary Figure S2). This Erb1 insertion area shows poor sequence conservation (Supplementary Figure S1) and will not be taken it into further consideration. Ytm1 residues interacting with Erb1 appear to cluster mostly on one side of the interaction surface (Figure 1D) although they do not show sequence continuity of interacting residues (Figure 1D, Supplementary Figure S2 and Supplementary Table S2).

According to the previous observations, a set of six peptides, summarized in Table 1, were selected derived from Erb1 sequence and Ytm1 (Figures 1E,F and Supplementary Figures S1, S2).

**TABLE 1:** Peptides used in this study. Residue numbering is referred to the *Chaetomium thermophilum* sequences.

Peptide	Sequence	Protein	Residues
P1	VWELLTGRQVW	Erb1	479–489
P2	QQTIFRGH	Erb1	445–452
P3	DWHPREPWCV	Erb1	780–789
P4	HDDWVSA	Ytm1	110–116
P5	AGMDRTV	Ytm1	194–200
P6	RGHANKV	Ytm1	385–391
Biot-P1	Biotin-VWELLTGRQVW	Erb1	479–489
Biot-P3	Biotin-DWHPREPWCV	Erb1	780–789
Biot-P6	Biotin-RGHANKV	Ytm1	385–391

### Peptide Competition Assay

**FIGURE 2:**
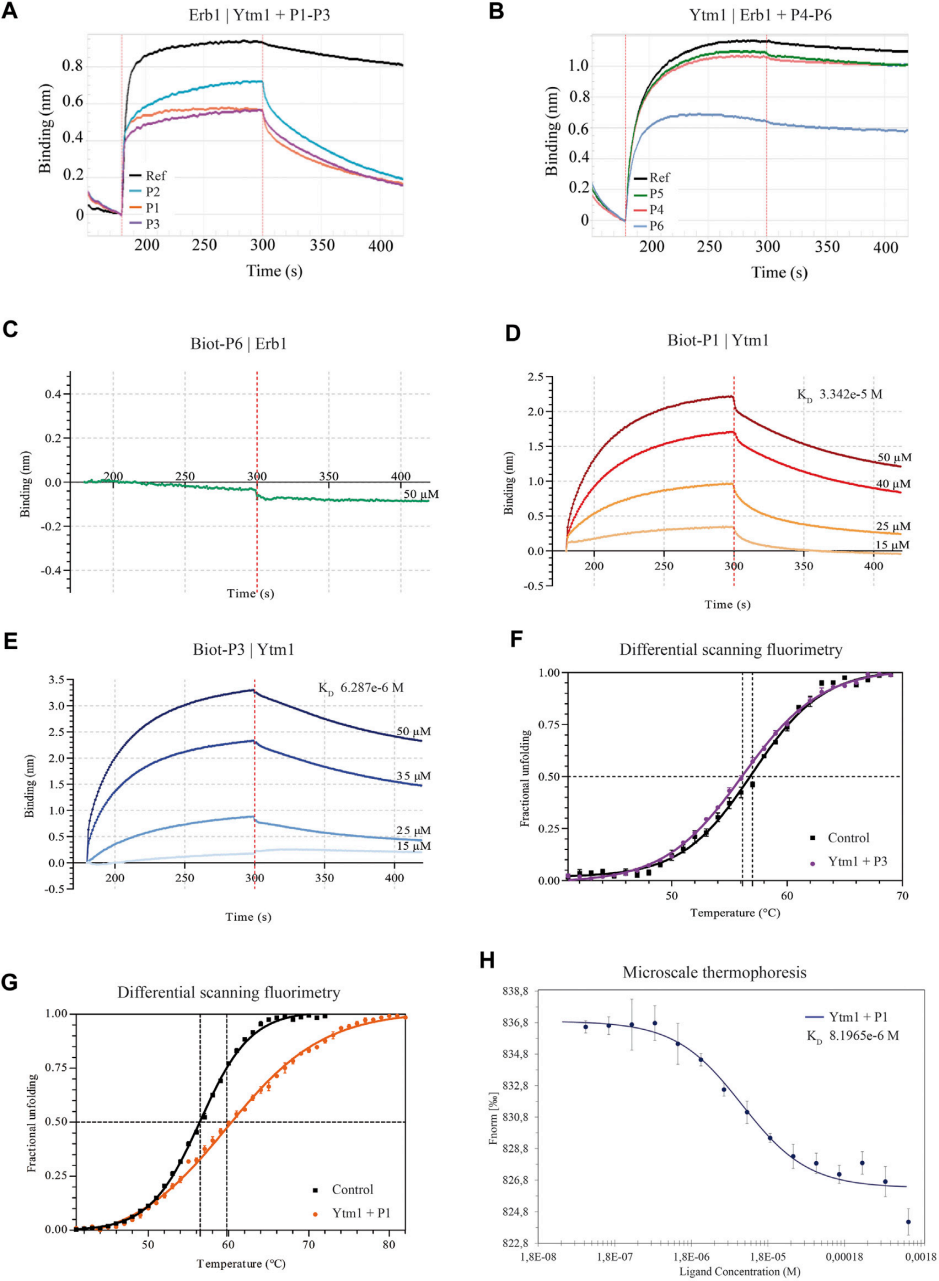
Binding properties of Erb1- and Ytm1-derived peptides to Ytm1 and Erb1, respectively. **(A)** Biolayer interferometry curves of preincubated peptides P1–3 with equimolar amounts of Ytm1 (5 µM). The ability to interfere with Erb1/Ytm1 complex formation (black line) is evaluated using immobilized Erb1 and Ytm1 as an analyte. The reference K_D_ for the Erb1/Ytm1 complex is 3.228e-8M. K_D_ decrease for preincubated Ytm1-P2 is less than one order of magnitude (Supplementary Table S3) whereas it is more than one order of magnitude for preincubated Ytm1-P1 and Ytm1-P3. **(B)** The equivalent competition BLI experiment preincubating equimolar concentrations of peptides P4–6 with 20 µM Erb1 and using immobilized Ytm1 as bait. K_D_ values of Erb1-P5 and Erb1-P4 are similar to the reference K_D_ (2.292e-7M) using only Erb1 as an analyte. K_D_ values of Erb1-P6 are almost one order of magnitude lower than the reference (Supplementary Table S3). **(C–E)** Biolayer interferometry curves using inmobilized biotynilated peptides. **(C)** Immobilized biot-P6 did not show binding when using Erb1 as analyte. **(D)** BLI Ytm1 binding curves obtained using immobilized biot-P1. **(E)** BLI Ytm1 binding curves using biot-P3 as bait. **(F)** Thermal shift curves obtained by differential scanning fluorimetry (DSF) of Ytm1 isolated (black line) and in the presence of 1 mM P3 peptide. **(G)** Similarly, DSF thermal shift curves of Ytm1 isolated (black line) and in the presence of 1 mM P1 peptide (orange). **(H)** Microscale thermoforesis binding curve for Ytm1 with peptide P1. Ytm1 was labeled using the Red-NHS second-generation dye for the binding affinity with the peptide P1 (NanoTemper Technologies). Fnorm indicates normalized fluorescence.

Biolayer interferometry interference assays were used to evaluate the ability of each peptide to interfere in the *in vitro* formation of the Erb1/Ytm1 complex. Erb1 was immobilized to a Ni-NTA biosensor and Ytm1 was used as analyte for testing direct interaction. The binding affinity is 3.2e-8M (Supplementary Table S3), similar to the previously reported values using microcalorimetry (Wegrecki et al., 2015). Peptides were evaluated for their ability to decrease in the Erb1/Ytm1 complex formation by preincubating the analyte with each peptide derived from the immobilized partner. Figures 2A,B show typical curves from the interference assay using biolayer interferometry. The black line depicts the reference curve obtained in the absence of peptide preincubation. Erb1-derived peptides were evaluated by their ability to decrease Erb1/Ytm1 complex affinity. Each P1-3 peptide was preincubated for half an hour at 4°C with an equimolar concentration of Ytm1 used as an analyte for binding with Erb1, previously immobilized to the Ni-NTA biosensor (Figure 2A). Peptides P1 and P3 showed a decrease of more than one order of magnitude (1.9 and 1.7e-7M respectively) with respect the Erb1/Ytm1 binding curve, in the absence of peptide. Similarly, an assay immobilizing Ytm1 to the Ni-NTA biosensor was used with Erb1 as analyte preincubated at equimolar concentrations with P4-6 Ytm1-derived peptides. Only peptide P6 showed a significant decrease (2.4e-6M) with respect to the reference Ytm1/Erb1 binding curve (Figure 2B).

### Direct Peptide Binding Assays

According to P1, P3, and P6 results, a new N-terminal biotynilated peptide synthesis was ordered using the same sequences (Biot-P1, Biot-P3, and Biot-P6, Table 1). Biotynilated peptides Biot-P1 and Biot-P3 were immobilized to the streptavidin biosensor and Ytm1 binding was measured. Similarly, Biot-P6 was immobilized to the streptavidin biosensor followed by Erb1 binding. As indicated in Figure 2C, we could not detect binding for Biot-P6/Erb1. Peptides Biot-P1 and Biot-P3 showed an affinity for Ytm1 in the mM range (Figures 2D,E). To further confirm the interaction a differential scanning fluorimetry assay was performed. A thermofluor assay of Ytm1 on its own and in the presence of 1 mM P3 did not show any significant thermal shift (Figure 2F) Similarly, no significant thermal shift in Ytm1 was observed in the presence of 1 mM P2 (Supplementary Figure S3). A shift of several degrees in the Tm was observed in the presence of 1 mM P1 indicating binding with Ytm1 (Figure 2G). Inconsistent results were obtained in DSF using Erb1 and a proper Tm could not be obtained so thermal shift could not be evaluated for P6. To further demonstrate the ability of P1 to interact with Ytm1, the binding was also monitored in free solution by the change in the thermophoresis of Ytm1 upon interaction with P1. Binding affinity determined using microscale thermophoresis showed a Kd in the mM range (8.2e-6M, Supplementary Table S3) consistent with affinities observed using interferometry and further confirming a direct interaction between P1 and Ytm1 (Figure 2H). Experimental limitations with maximum ligand concentration did not allow the calculation of binding stoichiometry. A tendency toward binding was also detected using MST with the P3 peptide at a maximum concentration of 1,580 µM but data quality would not allow a proper fitting for obtaining the K_D_ values (Supplementary Figure S4).

## Discussion

Erb1 surface appears to be more suitable for the selection of peptides targeting the complex, as compared to Ytm1 that, despite showing some degree of clustering (Figure 1D) reveals scattered interacting residues and fragmented regions (Supplementary Figure S2). In fact, we did not obtain clear experimental binding results for Ytm1-derived peptides. Apart from the peptides derived from Ytm1 selected in this study, additional regions that might have been considered are 224–229 that shows a rather continuous interaction with Erb1. It has, however, been initially discarded given the small differences in calculated ΔΔG upon *in vitro* alanine substitution with respect WT Ytm1 in the complex (Supplementary Table S2). An additional region includes V272 that shows reasonable ΔΔG values and continuity (S265-V272) of neighboring residues in *Chaetomium*. However, it is located in an evolutionary poorly conserved region (Supplementary Figure S2). Sequence elongation of the selected Ytm1-derived peptides might also provide better interaction results.

The combination of in silico hot spot analysis with contact analysis in the interface and residue conservation suggested an initial set of six peptides in the Erb1/Ytm1 interface. Three of them showed some degree of interference in the complex formation on a competition assay. Best candidates (P1, P3, and P6) were further tested immobilizing the peptide to the biosensor. We have observed some differences for peptide P6 in the competition assays with respect biotinylated peptide assay using biolayer interferometry. Biotin itself could be affecting the binding to Erb1; however, this is unlikely to be the case since the N-terminus of P6 is not involved in the interaction according to the structure of the Erb1/Ytm1 complex (Figure 1). An obvious difference is the immobilization of the peptide that might be impairing a proper conformation of P6 for the interaction with Erb1 to take place. Immobilized peptide in the biotynilated P1 experiment can also explain the slight difference observed in the Kds obtained by BLI and MST. Tm values for Erb1 using DSF could not be obtained. Moreover, several of the selected peptides did not show any effect on the interference biolayer interferometry assay. Alternative methodological approaches should be considered for the detection of low affinity interactions in BLI or proteins with potential hydrophobic exposed areas in DSF. We have been able to detect direct *in vitro* interactions of P1 and Biot-P1 with Ytm1 at a low mM affinity range indicating that at least for these conditions biolayer interferometry is a suitable methodological approach. MST also represented a good alternative for evaluating the interaction with initial binding detected for Ytm1-P3 and proper affinity determination for Ytm1-P1. Erb1 and Ytm1 form a part of the Nop7 complex which is a heterotrimeric complex. Assembly of the three subunits is required for the correct maturation of 60S ribosomal subunit. Once the Nop7 complex has exerted its molecular function it is sequentially removed from the pre60S subunit (Tang et al., 2008; Baßler and Hurt, 2019). Individual Nop7, Erb1, and Ytm1 will then reassemble to repeat the process with a new preribosome. It is at this stage when a peptide targeting the Erb1/Ytm1 interaction could play its interference role since the P1 peptide affinity shown for Ytm1 is lower than the one observed for the Erb1/Ytm1 complex.

Ribosome biogenesis has recently been accumulating growing attention as a potential new therapeutic target, since the observation that cell proliferation can be blocked by inhibition of new ribosomes production. Several RNA pol I inhibitors have been reported to date (Ferreira et al., 2020) and molecules like CX-3543, CX-5461, and BMH-21 are currently under investigation for treating cancer, as rapidly dividing cancer cells are particularly dependent on high levels of RNA pol I transcription. CX-5461 is phase I clinical trial in patients with advanced haematological cancers and breast cancer (Hilton et al., 2018; Khot et al., 2019). CX-3543 reached phase II clinical trial but was withdrawn due to bioavailability issues (Balasubramanian et al., 2011). While extremely promising, these compounds targeting RNA pol I are associated with additional activities, like DNA damage, which possibly contributes to its efficacy, toxicity profile, and resistance mechanisms. These observations suggest that it may be necessary to look for other points of intervention during this multistep process of ribosome maturation. One relevant question in this new field of targeting ribosome biogenesis is whether downstream specific targets would behave the same or differ to those RNA pol I transcription inhibitors currently developed. We had previously shown that altering the interaction surface between the ribosome assembly factors Erb1 and Ytm1 hinders cell proliferation in yeast. We have now developed a peptide derived from the Erb1 sequence capable of interacting with Ytm1 at a low mM range and interfere in the Erb1/Ytm1 complex formation negatively affecting this extensive and highly conserved protein–protein interaction. This result opens the possibility to investigate the *in vivo* action of RNA pol I downstream targets in the ribosome biogenesis process. Delivery methods like cell penetrating peptides or nanoparticles should be considered for internalization of the peptide into the cell in order to test future inhibitory strategies *in vivo*.

## Data Availability

The raw data supporting the conclusions of this article will be made available by the authors, without undue reservation.
