# Supplementary figures and images for: Peptides Targeting the Interaction Between Erb1 and Ytm1 Ribosome Assembly Factors
Source: Front Mol Biosci. 2021 Sep 1;8:718941. doi: 10.3389/fmolb.2021.718941 (PMC8440923; doi:10.3389/fmolb.2021.718941)

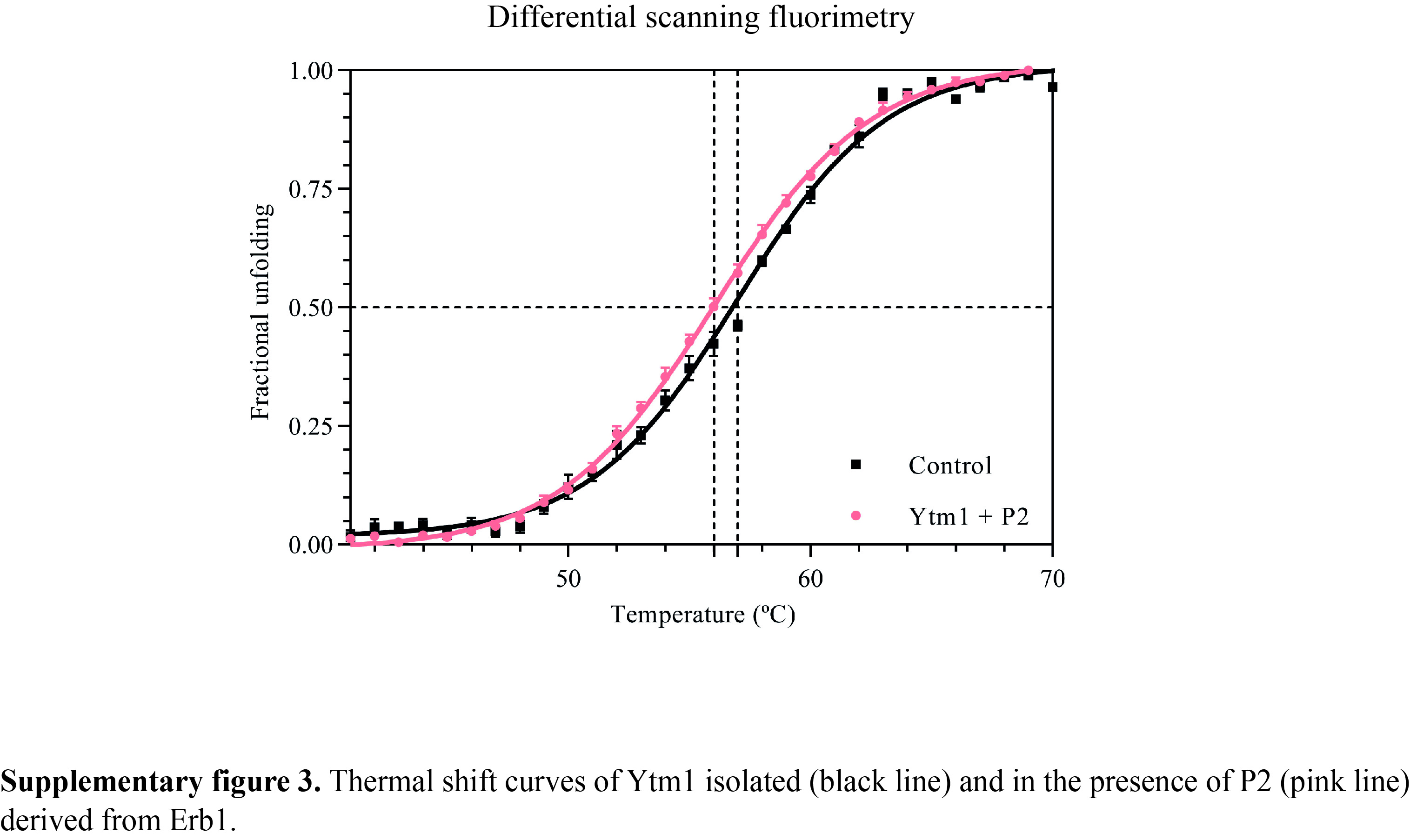

Supplement: Supplementary file 1 [file Image3.JPEG]

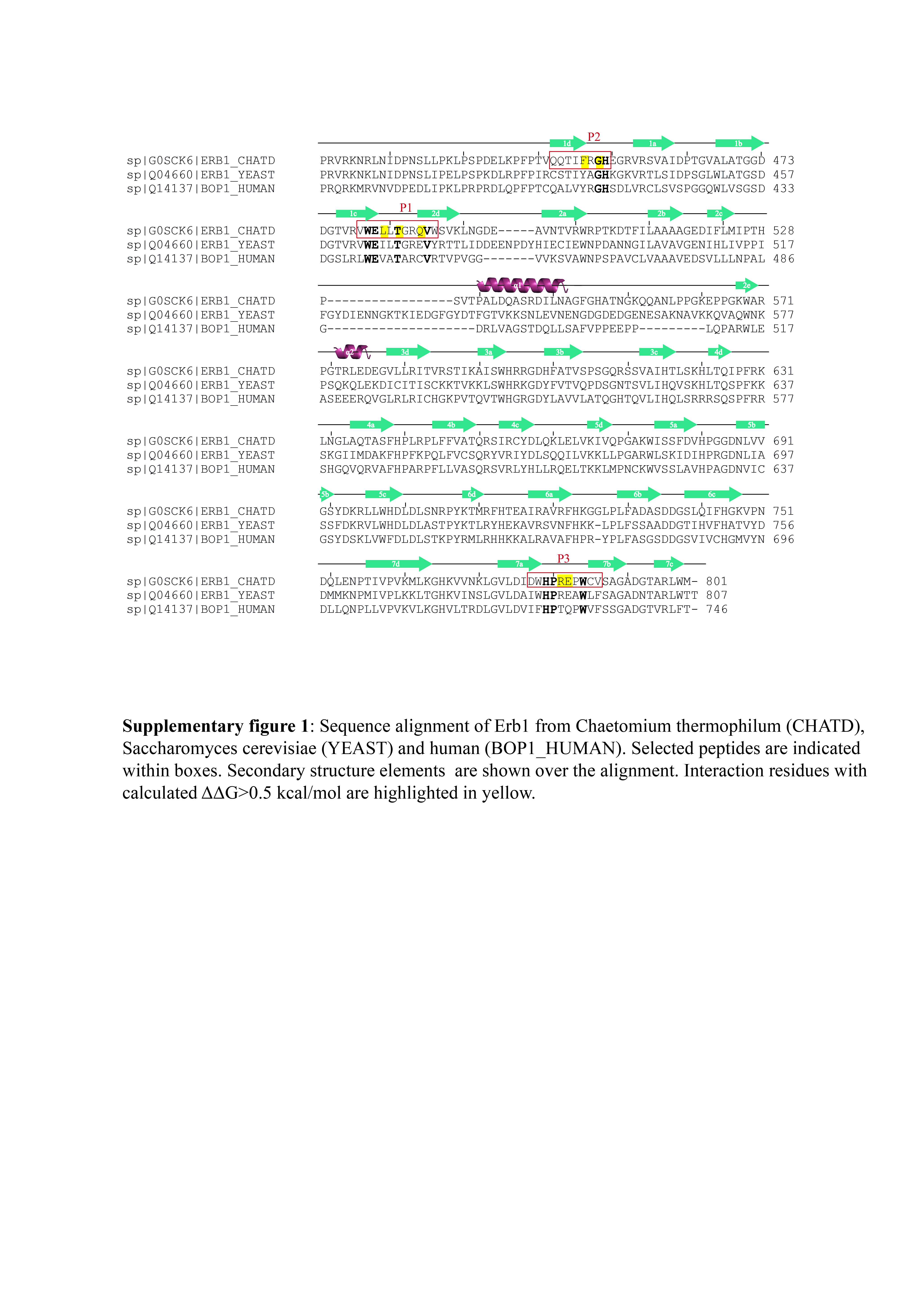

Supplement: Supplementary file 3 [file Image1.JPEG]

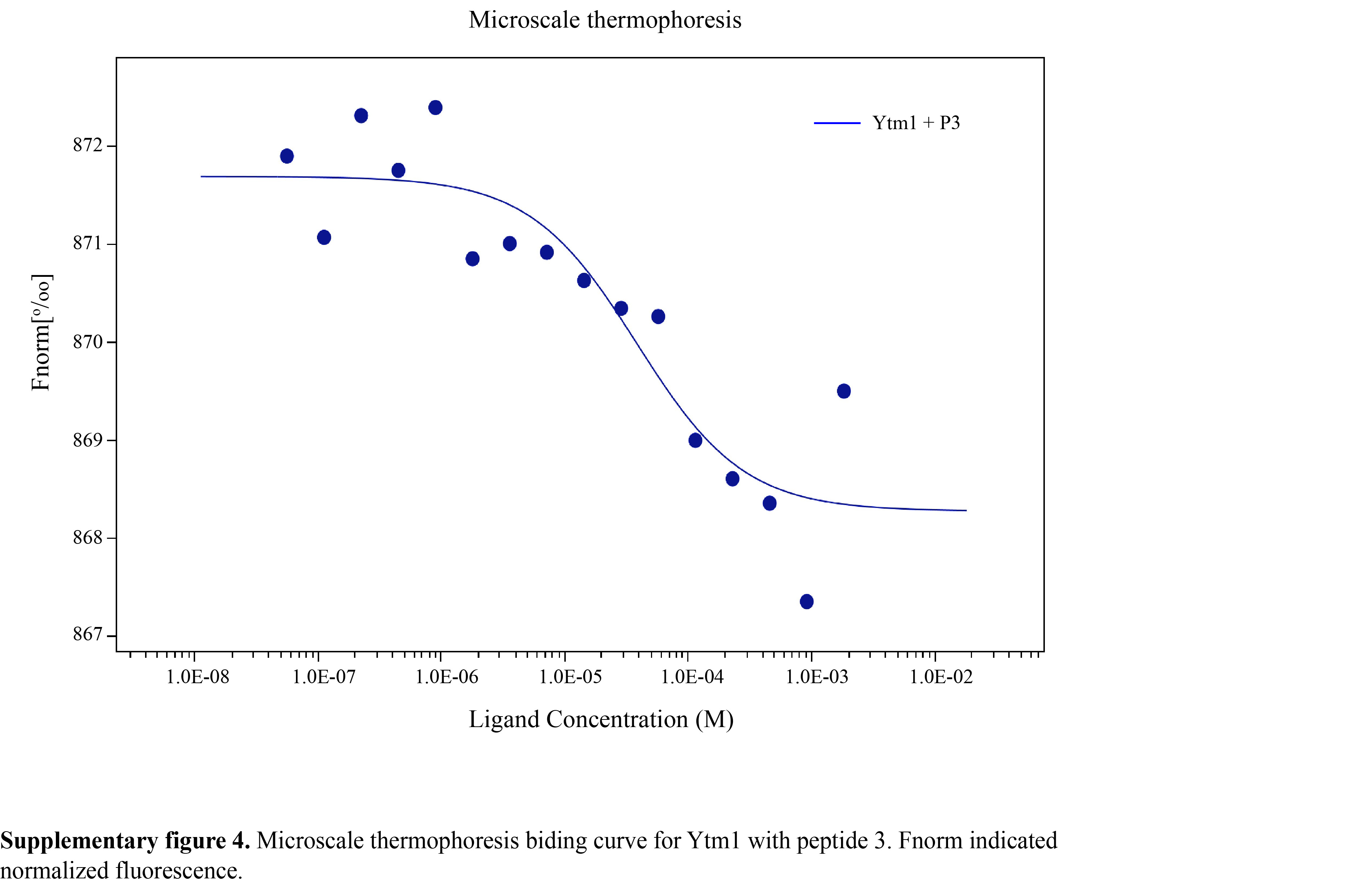

Supplement: Supplementary file 4 [file Image4.JPEG]

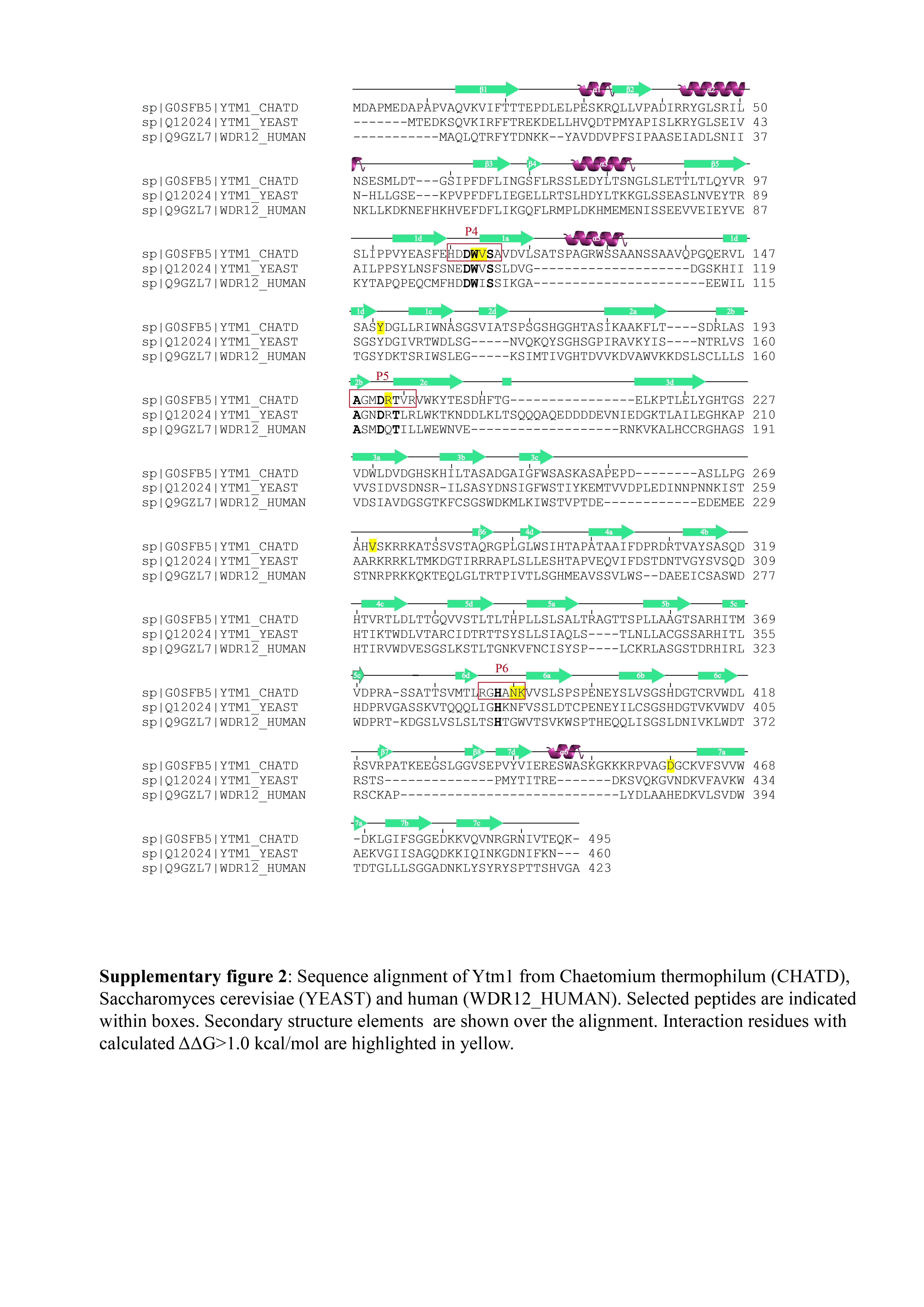

Supplement: Supplementary file 5 [file Image2.JPEG]
