# Supplementary material for: Peptides Targeting the Interaction Between Erb1 and Ytm1 Ribosome Assembly Factors
Source: Front Mol Biosci. 2021 Sep 1;8:718941. doi: 10.3389/fmolb.2021.718941 (PMC8440923; doi:10.3389/fmolb.2021.718941)
Supplement: Supplementary file 2 [file Table1.DOCX]

| Residue | ddGcal (kcal/mol) | Buriedness |
| --- | --- | --- |
| D436 | 0.11 | 3.78 |
| K439 | 0.08 | 2.5 |
| T443 | 0.18 | 4.82 |
| V444 | 0.47 | 6.43 |
| Q445 | 0.12 | 3.08 |
| Q446 | 0.48 | 10.55 |
| T447 | 0.42 | 8.52 |
| *I448 | 1.02 | 5.45 |
| *R450 | 0.53 | 3.03 |
| T464 | 0.14 | 4.89 |
| V466 | 0.42 | 4.27 |
| *E481 | 0.55 | 5.18 |
| L482 | 0.08 | 2.72 |
| *L483 | 0.65 | 1.89 |
| T484 | 0.36 | 6.08 |
| *R486 | 2.39 | 5.26 |
| Q487 | 0.14 | 4.96 |
| V488 | 0.4 | 6.43 |
| H528 | 0.09 | 3.85 |
| S530 | 0.09 | 3.21 |
| V531 | 0.2 | 5.38 |
| T532 | 0.43 | 7.4 |
| Q537 | 0.08 | 3.93 |
| H782 | 0.19 | 5.78 |
| *R784 | 0.54 | 3.77 |
| *E785 | 0.63 | 6.38 |
| W787 | 0.49 | 3.47 |
| R798 | 0.25 | 8.42 |
| L799 | 0.26 | 5.98 |
| M801 | 0 | 2.72 |

Supplementary table 1: In silico Erb1/Ytm1 complex alanine scanning on Erb1 as performed using DrugScore^PPI^. Calculated ΔΔG obtained for each Erb1 residue in the complex interface are indicated (ΔΔGcal= ΔG^ALA^complex - ΔG^WT^complex). Asterisks indicates residues with calculated ΔΔG>0.5 kcal/mol.
