# Supplementary material for: Peptides Targeting the Interaction Between Erb1 and Ytm1 Ribosome Assembly Factors
Source: Front Mol Biosci. 2021 Sep 1;8:718941. doi: 10.3389/fmolb.2021.718941 (PMC8440923; doi:10.3389/fmolb.2021.718941)
Supplement: Supplementary file 6 [file Table2.DOCX]

| Residue | ΔΔGcal (kcal/mol) | Buriedness |
| --- | --- | --- |
| *D112 | 1.69 | 8.78 |
| *W113 | 1.19 | 5.54 |
| *Y151 | 2.11 | 7.23 |
| D152 | 0.26 | 8.78 |
| S179 | 0.2 | 5.62 |
| K181 | 0.46 | 6.59 |
| M196 | 0.37 | 3.61 |
| D197 | 0.29 | 10.06 |
| *R198 | 1.07 | 6.1 |
| H224 | 0.22 | 7.59 |
| T225 | 0.13 | 3.63 |
| S227 | 0.16 | 4.62 |
| D229 | 0.4 | 7.49 |
| S265 | 0.13 | 4.62 |
| L266 | 0.21 | 3.67 |
| L267 | 0.08 | 2.72 |
| H271 | 0.05 | 0.99 |
| *V272 | 1.23 | 2.16 |
| T297 | 0.17 | 3.7 |
| Q318 | 0.27 | 1.99 |
| D319 | 0.35 | 12.56 |
| H320 | 0.42 | 9.52 |
| H340 | 0.08 | 2.86 |
| L343 | 0.23 | 3.67 |
| S363 | 0.22 | 4.72 |
| R365 | 0.59 | 5.31 |
| *N389 | 1.63 | 12.99 |
| *K390 | 1.34 | 6.36 |
| H409 | 0.28 | 6.71 |
| K426 | 0.07 | 2.5 |
| E427 | 0.09 | 3.22 |
| V456 | 0.65 | 2.23 |
| *D459 | 1.94 | 5.07 |
| K462 | 0.74 | 5.82 |
| F464 | 0.3 | 5.87 |
| E478 | 0.31 | 8.46 |

Supplementary table 2: In silico Erb1/Ytm1 complex alanine scanning on Ytm1 as performed using DrugScore^PPI^. Calculated ΔΔG obtained for each Ytm1 residue in the complex interface are indicated (ΔΔGcal= ΔG^ALA^complex - ΔG^WT^complex). Asterisks indicates residues with calculated ΔΔG>1.0 kcal/mol.
