# Supplementary material for: Peptides Targeting the Interaction Between Erb1 and Ytm1 Ribosome Assembly Factors
Source: Front Mol Biosci. 2021 Sep 1;8:718941. doi: 10.3389/fmolb.2021.718941 (PMC8440923; doi:10.3389/fmolb.2021.718941)
Supplement: Supplementary file 7 [file Table3.DOCX]

**BLI Erb1 | Ytm1 + P1-P3 - Affinity values**

| Protein-Peptide | K_D_ (M) | ka (1/Ms) | ka Error | kd (1/s) | kd Error | X^2^ | R^2^ |
| --- | --- | --- | --- | --- | --- | --- | --- |
| Ref Ytm1-Erb1 | **3.222e-8** | **5.35e4** | **7.06e2** | **1.724e-3** | **5.535e-5** | **0.3016** | **0.9698** |
| Ytm1-P2 | 3.557e-7 | 3.916e4 | 9.705e2 | 1.393e-2 | 2.215e-4 | 1.578 | 0.9603 |
| Ytm1-P1 | 1.902e-7 | 1.021e5 | 2.602e3 | 1.942e-2 | 2.272e-4 | 0.5205 | 0.9822 |
| Ytm1-P3 | 1.745e-7 | 7.094e4 | 1.739e3 | 1.238e-2 | 1.536e-4 | 0.6148 | 0.974 |

**BLI Ytm1 | Erb1 + P4-P6 – Affinity values**

| Protein-Peptide | K_D_ (M) | ka (1/Ms) | ka Error | kd (1/s) | kd Error | X^2^ | R^2^ |
| --- | --- | --- | --- | --- | --- | --- | --- |
| Ref Erb1-Ytm1 | **2.292e-7** | **5.24e3** | **3.87e1** | **1.201e-3** | **2.504E-5** | **0.04878** | **0.9985** |
| Erb1-P5 | 2.825e-7 | 5.857e3 | 5.321e1 | 1.655e-3 | 4.406E-5 | 0.0926 | 0.9964 |
| Erb1-P4 | 2.966e-7 | 5.345e3 | 4.358e1 | 1.585e-3 | 1.704e-5 | 0.04445 | 0.9982 |
| Erb1-P6 | 2.439e-6 | 4.384e3 | 2.224e2 | 1.069e-2e | 2.341e-4 | 0.1423 | 0.9815 |

**BLI Biotin-peptide - Affinity values**

| Peptide-protein | K_D_ (M) | ka (1/Ms) | ka Error | kd (1/s) | kd Error | X^2^ | R^2^ |
| --- | --- | --- | --- | --- | --- | --- | --- |
| Biot-P1 | 3.342e-5 | 3.865e2 | 6.967e1 | 1.292e-2 | 5.954e-4 | 16.02 | 0.9852 |
| Biot-P3 | 6.287e-6 | 8.294e2 | 2.496e1 | 5.215e-3 | 1.941e-4 | 9.07 | 0.9962 |

**Microscale Thermoforesis Ytm1 + P1 - Dataset overview**

| Target name | Ytm1 (labelled Lys) |
| --- | --- |
| Target concentration | 5 nM |
| Ligand name | P1 |
| Ligand Concentration | 1.23E+3 µM to 0.0751 µM |
| n | 3 |
| Excitation Power | 100% |
| MST Power | 60% |
| KD | 8.1965E-6 |
| KD confidence | [5.1079E-06 – 1.3152E-05] |
| Response Amplitude | 10.582501 |
| Std. Error of Regression | 0.80869066 |
| Reduced X^2^ | 1.1260899 |
| Signal to Noise | 14.134462 |
